# Diagnostic analysis of new quantitative parameters of low-dose dynamic myocardial perfusion imaging with CZT SPECT in the detection of suspected or known coronary artery disease

**DOI:** 10.1007/s10554-020-01962-x

**Published:** 2020-09-10

**Authors:** Zekun Pang, Jiao Wang, Shuai Li, Yue Chen, Xiaojie Wang, Jianming Li

**Affiliations:** Nuclear Medicine Department, Tianjin Medical University Clinical Cardiovascular Institute, TEDA International Cardiovascular Hospital, Tianjin, 300457 China

**Keywords:** Cadmium zinc tellurium (CZT), Single photon emission computed tomography (SPECT), Myocardial perfusion imaging (MPI), Myocardial flow reserve (MFR), Relative flow reserve (RFR)

## Abstract

The goal of this study is to explore and evaluate the diagnostic values of myocardial blood flow (MBF), myocardial flow reserve (MFR) and relative flow reserve (RFR) obtained with low-dose dynamic CZT SPECT for patients with suspected or known coronary artery disease (CAD). Fifty-seven consecutive patients who underwent low-dose dynamic CZT SPECT and CAG were enrolled. MBF, MFR and RFR were calculated on the vessel level with dedicated quantitative software, and the difference and correlation of each parameter was compared according to the reference standard of stenosis ≥ 50% or ≥ 75% on CAG, respectively. ROC curves were made by stress MBF (sMBF), rest MBF (rMBF), MFR and RFR. The optimal cut-off values and corresponding diagnostic efficacy were obtained and compared with each other. Results indicated that when stenosis ≥ 50% or ≥ 75% on CAG was used as the reference standard at the vessel level, there was no statistically significant difference in rMBF between the negative group and the positive group (P > 0.05), and the sMBF and MFR in positive groups were significantly lower than that in the negative group (all P < 0.05). There was a moderate to significant correlation between sMBF and MFR, sMBF and RFR, MFR and RFR (all P < 0.0001). These results indicate that low-dose dynamic CZT SPECT imaging can easily obtain the sMBF, MFR and RFR, and there is a good correlation among the three parameters, which has a certain diagnostic value for patients with suspected or known CAD, and is a useful supplement to the conventional qualitative or semi-quantitative diagnostic methods.

## Introduction

Myocardial perfusion imaging (MPI) with single photon emission computed tomography (SPECT) is an important non-invasive imaging diagnostic tool in diagnosis, risk stratification, prognosis and efficacy evaluation for coronary artery disease (CAD) [1–5]. The conventional SPECT MPI evaluates the presence, extent and degree of myocardial ischemia and/or infarction usually through visual observation or semi-quantitative parameters, such as summed stress score (SSS), summed rest score (SRS), summed different score (SDS) and total perfusion defect (TPD), etc., and there have been many researches on this field [6, 7].

In recent years, the “milestone” progress of SPECT is the advent and clinical application of cardiac-dedicated SPECT equipped with cadmium-zinc-tellurium (CZT) of semi-conductor detectors, which has greatly improved the performance of SPECT equipment [8–10]. CZT SPECT not only greatly improves the detection sensitivity and spatial resolution, but also reduces the single dose of injected radiopharmaceuticals, thereby reducing the radiation dose for the patients. Meanwhile, because the significantly improved time resolution of the detection and the projection data in the 180° range from the left anterior inclination 45° to the left posterior inclination 45° can be acquired at the same time without detector rotation around the body, realizing the fast and dynamic scan, which makes CZT SPECT convenient for quantitative measurement of myocardial blood flow (MBF). Therefore, CZT SPECT can not only achieve low-dose MPI imaging, but also provide some new parameters for quantitative analysis of absolute MBF through fast dynamic tomography, such as stress or rest myocardial blood flow (sMBF or rMBF), myocardial flow reserve (MFR) and relative flow reserve (RFR). Quantitative analysis has obvious advantages over visual observation or semi-quantitative analysis, especially for the left main (LM) and/or 3-vessels diseases, which improves the detective sensitivity and avoids missed diagnosis or underestimation [11–14].

In the past, quantitative analysis of MPI mainly relied on positron emission computed tomography/computed tomography (PET/CT), but due to the expensive price and the limitation of the availability of positron perfusion imaging agents, it has not been widely used in clinical practice. With the popularization of cardiac-dedicated CZT SPECT, the quantitative analysis of MPI is highly expected again, and recently, some comparative studies [15, 16] have shown that it correlates well with the quantitative parameters measured by PET/CT. However, there are still relatively few studies on quantitative parameters obtained by CZT SPECT MPI in the diagnosis of CAD until now. Furthermore, according to literature retrieval, we discovered that the comparative analysis among MBF, MFR and RFR has not been reported yet. In this article, we intends to explore and compare the diagnostic values of MBF, MFR and RFR in patients with suspected or known CAD through quantitative analysis with low-dose dynamic CZT SPECT MPI.

## Methods

### Study Population

Patients with suspected or known CAD who underwent low-dose dynamic CZT SPECT MPI were continuously enrolled. Inclusion criteria: (1) The age is between 18 and 79 years old; (2) Have the invasive CAG data within 3 months before and after MPI examination, no revascularization treatment during the period; (3) Suitable for adenosine triphosphate (ATP) drug-stressed MPI and can tolerate the dynamic SPECT imaging process; (4) Signed the informed consent form; (5) The imaging data passed the quality control. Exclusion criteria: Highly unstable angina pectoris, old myocardial infarction, post-operative revascularization, atrioventricular block of degree II and above, sick sinus syndrome (except those who have a pacemaker), COPD (including asthma, bronchiectasis, emphysema, pulmonary fibrosis, etc.), severe hypotension (systolic blood pressure < 90 mmHg), severe mitral or aortic valve disease, cardiomyopathy (dilated, hypertrophic cardiomyopathy, etc.), CAG negative, but has clinical diagnosis of highly suspected coronary microvascular dysfunction (CMD) (such as syndrome X and microvascular angina) or coronary artery spasm, patients who fail to complete dynamic acquisition or complete dynamic acquisition but the image quality is not satisfactory, pregnant or lactating women. All patients were given informed consents and this study was approved by the Ethics Committee of TEDA International Cardiovascular Hospital(Tianjin, China).

### Acquisition of CZT SPECT imaging

The device was a cardiac-dedicated SPECT (NM530c, GE Healthcare, Milwaukee, WI, USA), equipped with a CZT detector and the radionuclide imaging agent was ^99m^Tc-MIBI. The technetium was provided by Beijing Senke Pharmaceutical Co., Ltd. or Tianjin HTA Isotope Pharmaceutical Co., Ltd. The MIBI was provided by Jiangyuan Pharmaceutical Factory of Jiangsu Atomic Energy Research Institute. The labeled radiochemical purity was ≥ 95%. Preparation for patients: within 24 h before the examination, strictly prohibit patients to drink coffee, tea, or any food containing caffeine and theophylline, and stop taking conventional medications for cardiovascular disease.

The acquisition used a “single-day” or “two-day” protocol: the “single-day” protocol was patients underwent CZT imaging with the administration of 185–296 MBq of ^99m^Tc-MIBI during rest and three times dose of that at stress. ATP-stress imaging was performed 1–4 h after rest imaging and the method refer to the literature [17]. The “two-day” protocol injected the same dose of imaging agent at rest and stress was 370–555 MBq. Drank 350–500 mL of water before each collection on the machine. Rest imaging: pre-injected imaging agent 18.5–37 MBq (for pre-position imaging); after pre-positioning, started dynamic acquisition (list mode acquisition for 10 min), 10 s after started the program, “blous” injected imaging agent through the embedded vein channel within 5 s, and conventional rest gated tomography was performed 40–60 min after the dynamic acquisition. Stress imaging: After the patient's heart is pre-positioned, the imaging agent was injected at the peak of ATP-stress (at the 3rd min) and the injection method was same to the resting imaging. Continuous acquisition in list mode for 10 min, and then, after an interval of 15–30 min, the conventional stress gating tomography was carried out. General gated acquisition parameters: eight frames/cardiac cycle, heart rate window width ± 15%, and an energy window centered on the photopeak of 140 keV ± 10%. “Single-day” protocol’s rest and stress imaging were collected for 6 min and 4 min, respectively, and “two-day” protocol’s rest and stress imaging were both collected for 4 min. All patients needed to collect CT (NM690, GE company, USA) before SPECT imaging for attenuate correction data: the voltage was 120 kV, the current was 20 mA, the scanning range was from the lung tip to the middle and lower part of the liver.

### Imaging analysis and judgment criteria

All dynamic list mode data was transferred to the MyoFlowQ (Beijing Bailingyun Bio-Pharmaceutical Co., Ltd.) workstation and automatically reframed into a series of dynamic images: 10 s × 10 frames, 20 s × 5 frames, 60 s × 2 frames, 280 s × 1 frame; then called the CT attenuation correction data to complete the fusion alignment, axial adjustment, attenuation and scattering correction of the CT and SPECT images; automatically or manually adjusted the region of interest (ROI) of the blood pool curve input function and myocardial basal position, the dynamic curve and fitting curve of LV blood pool and LV myocardium were finally obtained. From this, rMBF and sMBF of LV 3-vessel regions were gained, and then, the MFR of the 3-vessel regions was calculated (MFR = sMBF/rMBF). Afterwards, the product of resting systolic blood pressure and heart rate, that is, RPP (rate pressure product), was used to correct rMBF. The RFR of a vascular region in the bullseye was defined as the ratio of the average sMBF of the myocardial segment within the vascular perfusion region of that branch to the sMBF of the normal reference myocardial region [18]. The routine reconstructions for gated MPI were performed with the software of QPS + QGS (Cedars Sinai medical center, Los Angeles, USA). The 3-vessels of the LV included the left anterior descending (LAD), the left circumflex (LCX), and the right coronary artery (RCA).

### Determination of vascular stenosis on CAG

Standard Judkins method was used for CAG. Two cardiologists with more than 3 years of interventional experience assessed the stenosis of coronary arteries with diameter ≥ 2 mm (visual assessment, consultation when there was a difference of opinion), and using stenosis ≥ 50% or ≥ 75% as the criteria for judging the positive.

### Statistical analysis

Used stenosis ≥ 50% and ≥ 75% on CAG as the reference standard for diagnosing CAD, the rMBF, sMBF, MFR, and RFR values of each vascular region in each group were obtained at the vessel level, and the following statistical analysis was performed: (1) compared the differences of quantitative parameters between the two standards; (2) calculated the correlation of quantitative parameters between two standards; (3) made ROC curves for rMBF, sMBF, MFR, and RFR under two standards, to obtain the best cut-off value and the corresponding diagnostic efficacy for obstructive CAD and compared them with each other; (4) excluded the patients with 3-vessels disease in each group (each major branch has at least one stenosis ≥ 50%) and repeated the process (3). All data were processed using IBM SPSS 17.0; measurement data were expressed as x ± sd, and comparison was performed using *T* test; the relationship between quantitative parameters was analyzed by correlation. P < 0.05 was considered statistically significant.

## Results

Fifty-seven patients with suspected or known CAD were included in this study, and their characteristics were shown in Table 1. The patients’ mean age was 62.1 ± 7.7 years, mean stature was 167 cm ± 8 cm, mean weight was 74.3 ± 11.3 kg, and 56.1% (n = 32) of the study population was male.

**Table 1:** Characteristics of the study population

Characteristics	N = 57 (%)
Male, n (%)	32 (56.1)
Female, n (%)	25 (43.9)
Negative (stenosis < 50% on CAG), n (%)	16 (28.1)
1-vessel CAD (stenosis ≥ 50% on CAG), n (%)	14 (24.6)
2-vessels CAD(stenosis ≥ 50% on CAG), n (%)	12 (21.1)
3-vessels CAD(stenosis ≥ 50% on CAG), n (%)	15 (26.3)
Hypertension, n (%)	37 (64.9)
Hyperlipidemia, n (%)	25 (43.9)
Diabetes, n (%)	9 (15.8)
Smoking, n (%)	19 (33.3)
Family history of CAD, n (%)	18 (31.6)

**Fig. 1:**
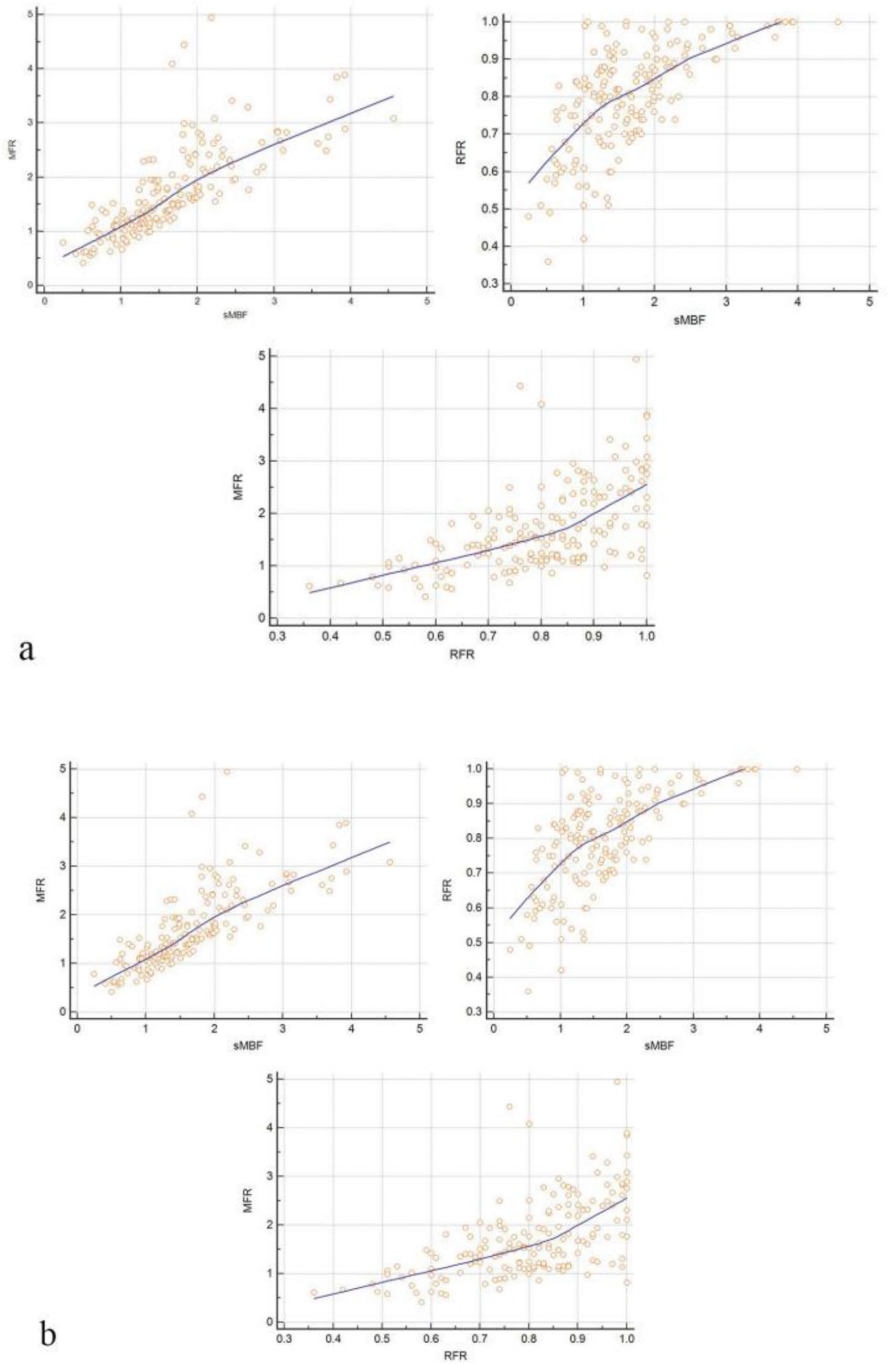
At vessel level (n = 171), the correlation scatter diagram among sMBF, MFR and RFR (the correlation among the three parameters was moderate or above, p < 0.001). **a** Stenosis ≥ 50%, the upper left shows the correlation scatter diagram of sMBF and MFR, the upper right shows the correlation scatter diagram of sMBF and RFR, and the lower shows the correlation scatter diagram of MFR and RFR; **b** stenosis ≥ 75%, the upper left shows the correlation scatter diagram of sMBF and MFR, the upper right shows the correlation scatter diagram of sMBF and RFR, and the lower shows the correlation scatter diagram of MFR and RFR

As shown in Table 2, when stenosis ≥ 50% or ≥ 75% on CAG was used as the reference standard at the vessel level, there was no statistically significant difference in rMBF between the negative group and the positive group (P > 0.05). Conversely, the sMBF and MFR in positive groups were both significantly lower than that in the negative group under two reference standards, and the difference was statistically significant (P < 0.05). Similarly, sMBF and MFR, sMBF and RFR, MFR and RFR all showed moderate to significant correlations. The correlation coefficients of the same group’s quantitative parameters were all similar under the two reference standards (r = 0.7611 and r = 0.6430, respectively, P < 0.0001) and the scatter diagram were shown in Fig. 1.

**Table 2:** Quantitative parameters and comparison among groups under two reference standards (at vessel level, n = 171)

Reference standard	rMBF (ml/g/min)		Statistic values	sMBF (ml/g/min)		Statistic values	MFR		Statistic values	RFR		Statistic values
	Negative group	Positive group		Negative group	Positive group		Negative group	Positive group		Negative group	Positive group	
≥ 50%	0.98 ± 0.25	0.93 ± 0.24	t = 1.40	2.03 ± 0.82	1.26 ± 0.50	t = 7.49	2.06 ± 0.83	1.34 ± 0.59	t = 6.51	0.80 ± 0.13	0.72 ± 0.14	t = 4.20
			P = 0.69			P = 0.00*			P = 0.00*			P = 0.00*
≥ 75%	1.03 ± 0.24	0.95 ± 0.25	t = 1.94	1.89 ± 0.79	1.15 ± 0.48	t = 7.61	1.93 ± 0.82	1.25 ± 0.56	t = 6.26	0.83 ± 0.13	0.74 ± 0.14	t = 4.21
			P = 0.05			P = 0.00*			P = 0.00*			P = 0.00*

*P < 0.05

*P < 0.05

**Fig. 2:**
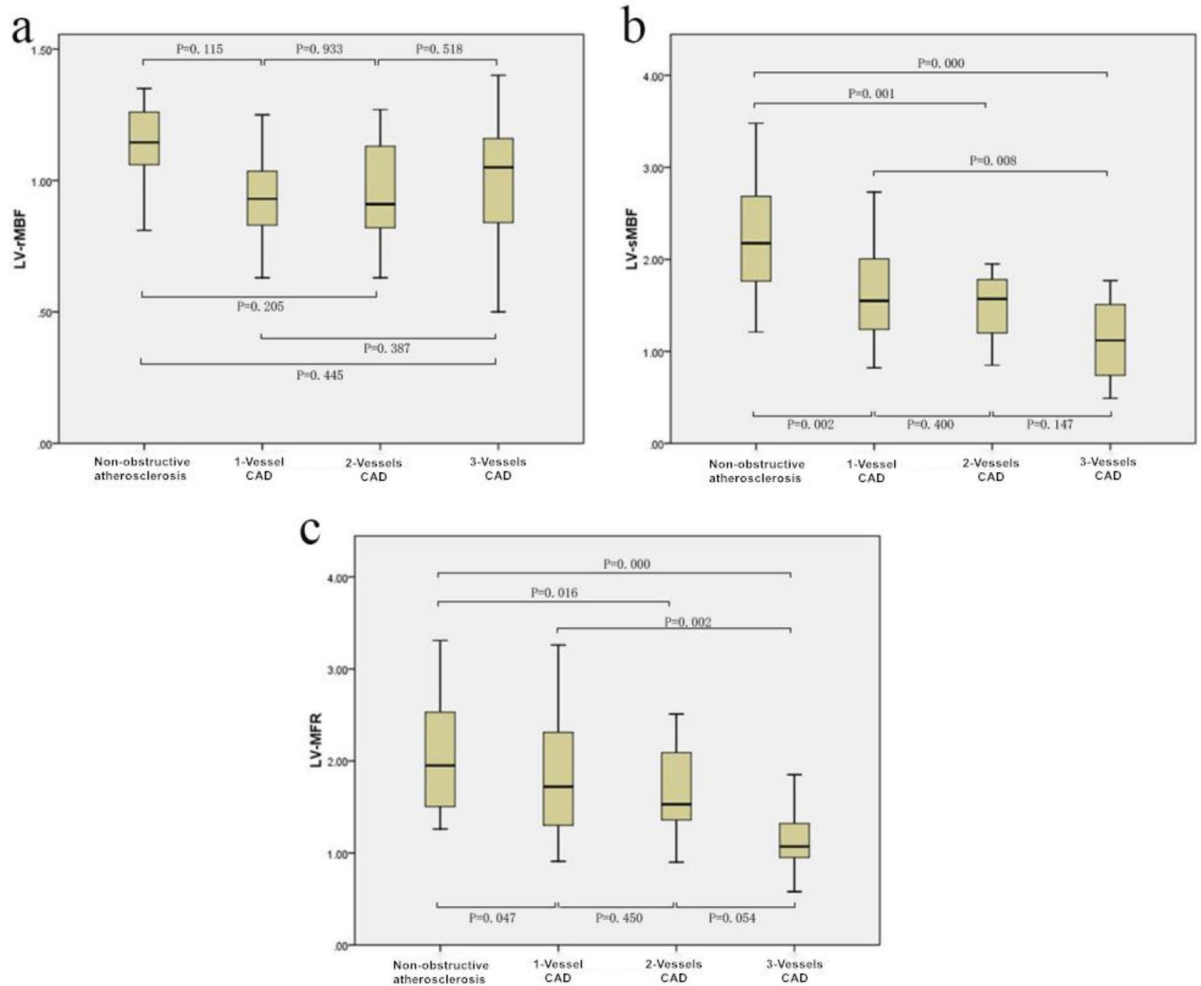
The comparison of rMBF, sMBF and MFR in four groups of non-obstructive atherosclerosis group, 1-vessel CAD group, 2-vessels CAD group and 3-vessels CAD group (at case level, with stenosis ≥ 50% as the reference standard). **a** distribution and comparison of rMBF. **b** distribution and comparison of sMBF. **c** distribution and comparison of MFR

According to the standard of stenosis ≥ 50% of LAD, LCX and RCA on CAG, the patients were divided into 4 groups: non-obstructive atherosclerosis group (negative stenosis in all vessels), 1-vessel CAD group, 2-vessels CAD group and 3-vessels CAD group. The average and standard deviation of LV-rMBF, LV-sMBF and LV-MFR in each group were calculated. (1) non-obstructive atherosclerosis group: the values were 1.08 ± 0.27 ml/g/min, 2.33 ± 0.80 ml/g/min and 2.28 ± 0.86 ml/g/min, respectively. (2) 1-vessel CAD group: the values were 0.95 ± 0.17 ml/g/min, 1.67 ± 0.54 ml/g/min and 1.82 ± 0.67 ml/g/min, respectively. (3) 2-vessels CAD group: the values were 0.95 ± 0.21 ml/g/min, 1.47 ± 0.42 ml/g/min and 1.62 ± 0.54 ml/g/min, respectively. (4) 3-vessels CAD group: the values were 1.02 ± 0.07 ml/g/min, 1.12 ± 0.39 ml/g/min and 1.11 ± 0.32 ml/g/min, respectively. The comparison of LV-rMBF, LV-sMBF and LV-MFR in each group was shown in Fig. 2. According to Fig. 2, there was no statistical difference between any two groups of LV-rMBF (P > 0.05), and on the contrary, there was a statistically significant difference between most paried groups for LV-sMBF and LV-MFR (except for 1-vessel CAD group vs 2-vessels CAD group and 2-vessels CAD group vs 3-vessels CAD group) (P < 0.05).

**Fig. 3:**
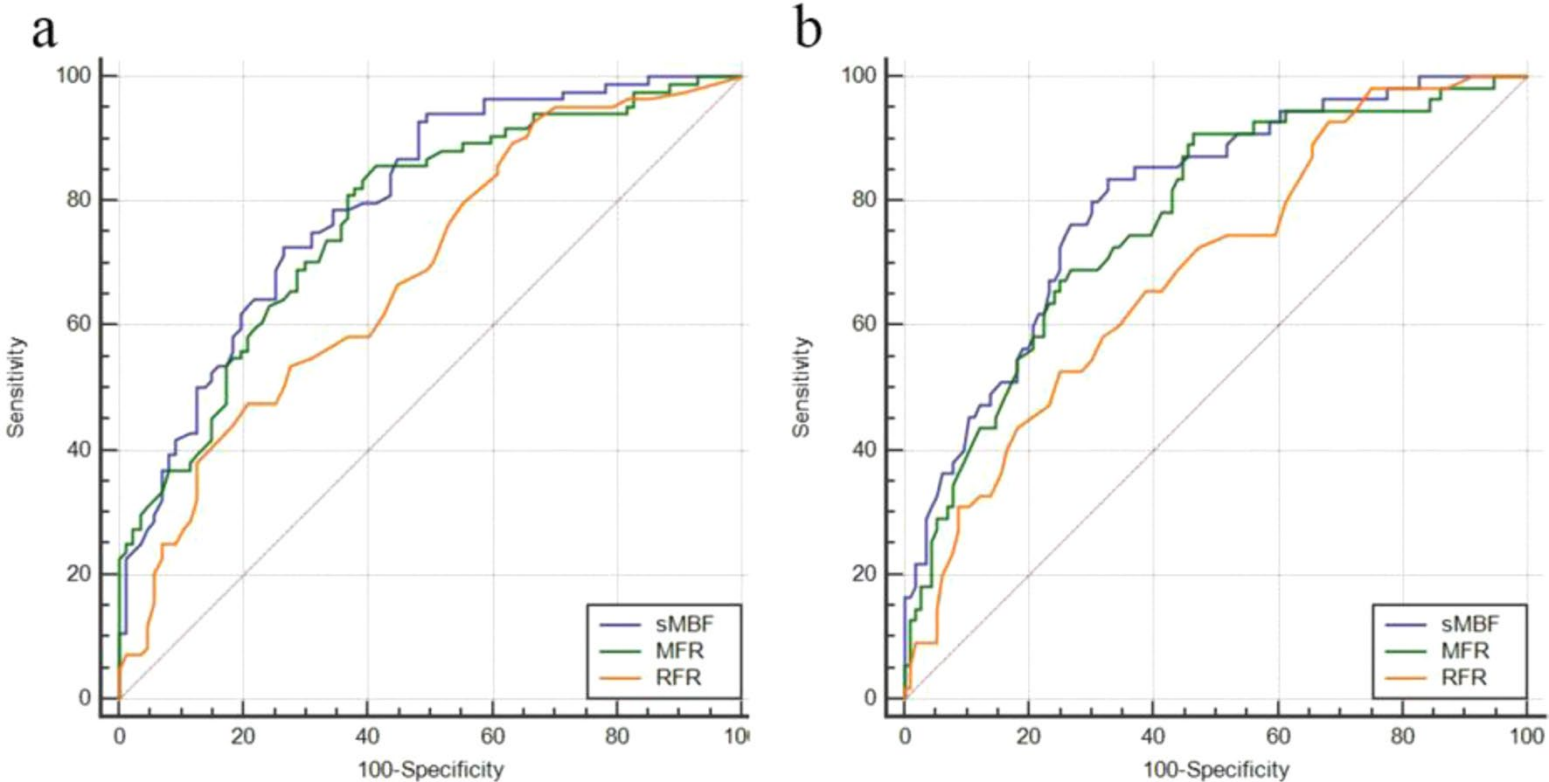
At vessel level, the ROC curve of sMBF, MFR and RFR under two reference standards. **a** Stenosis ≥ 50%, the AUC comparison of sMBF and RFR has statistical difference (P < 0.05), and the AUC comparison of sMBF and MFR, MFR and RFR has no significant difference (P > 0.05). **b** Stenosis ≥ 75%, the AUC comparison of sMBF and RFR, MFR and RFR has statistical difference (P < 0.05), and the AUC comparison of sMBF and MFR has no significant difference (P > 0.05)

The relevant parameters of the ROC curve of each quantitative parameter under two reference standards were shown in Table 3. With stenosis ≥ 50% as the reference standard, the AUC comparison of sMBF and MFR had no significant statistical difference: Z = 1.215, P = 0.2244; the AUC comparison of sMBF and RFR had statistical difference: Z = 3.003, P = 0.0027; and the AUC comparison of MFR and RFR had no significant statistical difference: Z = 2.282, P = 0.0225. Based on the optimal cut-off values of sMBF and MFR determined in Table 3, the correct rates for the detection of 3-vessels CAD at the case level were 100% (15/15) and 73.3% (11/15), respectively. With stenosis ≥ 75% as the reference standard, the AUC comparison of sMBF and MFR had no significant statistical difference: Z = 1.471, P = 0.1414; the AUC comparison of sMBF and RFR had statistical difference: Z = 2.896, P = 0.0038; and the AUC comparison of MFR and RFR had statistical difference: Z = 1.982, P = 0.0475. The corresponding ROC curve was shown in Fig. 3.

**Table 3:** ROC curve parameters of quantitative parameters under two reference standards (at vessel level, n = 171)

Reference standard	Parameters	AUC	95% CI	Youden index	Optimal cut-off values	SN (%)	SP (%)	Z	P values
≥ 50%	sMBF	0.798	0.730–0.855	0.4618	1.48 ml/g/min	72.62	73.56	8.939	< 0.0001
	MFR	0.772	0.720–0.833	0.4433	1.77	85.70	58.6	7.649	< 0.0001
	RFR	0.685	0.609–0.753	0.2693	0.75	47.62	79.31	4.569	< 0.0001
≥ 75%	sMBF	0.801	0.733–0.858	0.5088	1.48 ml/g/min	83.64	67.24	8.563	< 0.0001
	MFR	0.770	0.699–0.831	0.4436	1.76	90.91	53.45	7.056	< 0.0001
	RFR	0.690	0.614–0.758	0.2773	0.75	52.73	75.00	4.480	< 0.0001

**Fig. 4:**
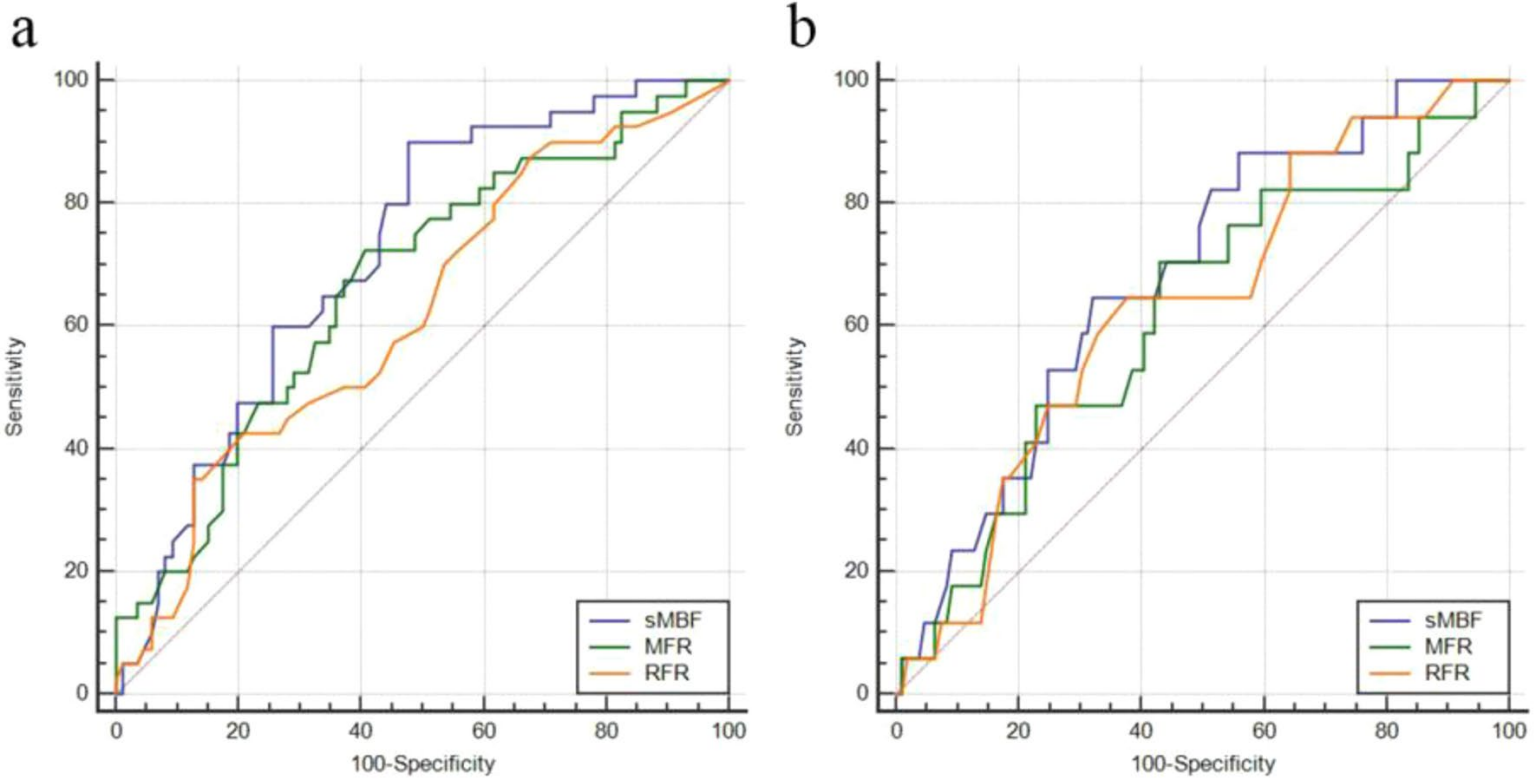
At vessel level (exclude patients with 3-vessels lesions), the ROC curve of sMBF, MFR and RFR under two reference standards. **a** stenosis ≥ 50% **b** stenosis ≥ 75%. The AUC comparison in (**a**) and (**b**) has no statistical difference (P > 0.05)

In order to eliminate the possible effect of 3-vessel CAD on RFR, the AUC comparison of the quantitative parameters under two reference standards was measured again after excluding the patients with 3-vessels CAD. After excluding data from patients with 3-vessels CAD, using stenosis ≥ 50% as the reference standard, the AUC comparison results of sMBF and MFR: Z = 1.565, P = 0.1177; the AUC comparison results of sMBF and RFR: Z = 1.753, P = 0.0796; and the AUC comparison results of MFR and RFR: Z = 0.813, P = 0.4164. There was no statistical difference in these results. Similarly, after excluding data from patients with 3-vessels CAD, using stenosis ≥ 75% as the reference standard, the AUC comparison results of sMBF and MFR: Z = 1.686, P = 0.0917; the AUC comparison results of sMBF and RFR: Z = 0.605, P = 0.5449; and the AUC comparison results of MFR and RFR: Z = 0.239, P = 0.8168. There was no statistical difference in these results. The corresponding ROC curve is shown in Fig. 4.

**Fig. 5:**
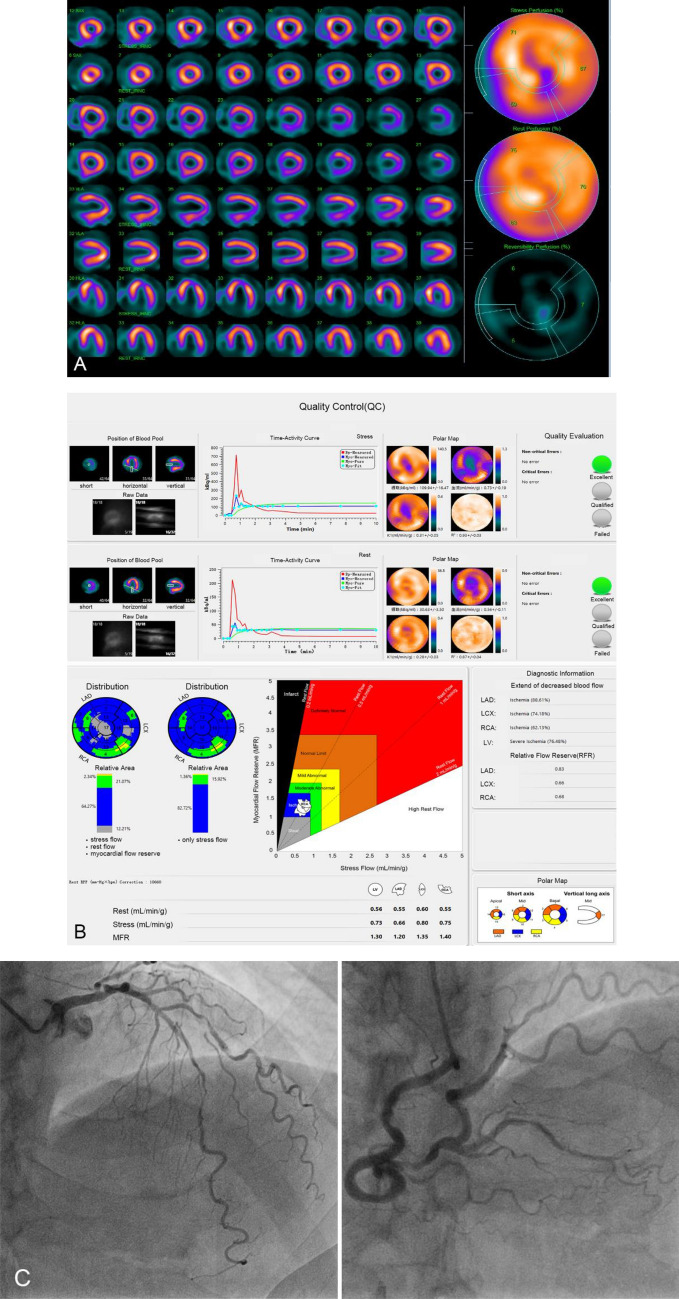
A 67-year-old man with untypical angina, high blood pressure and smoking history, without old myocardial infarction, PCI history, hyperlipidemia or diabetes. Routine gated MPI with ATP stress and rest imaging showed small area of ischemia in anterior and apical segments (**a**). Quantification analysis (**b**) showed MFR in LAD, LCX, RCA and the whole ventricle (LV) was significantly decreased, as well as RFR in LAD, LCX and RCA. Invasive CAG (**c**) showed diffuse stenoses in LAD and LCX, the worst stenosis was 90%, and multiple stenoses was showed in PDA and PLV of RCA, the worst stenosis was nearly 90%, and there was no stenosis in LM. The patients finally underwent coronary artery bypass grafting (CABG) surgery. **a** Routine ATP stress plus rest serial tomographic images. **b** Quantification analysis with MyoFlowQ for this patient: including quality control for dynamic data (upper) and quantitative information by this software. **c** Invasive CAG: diffuse severely stenotic lesions in LAD and, multiple stenotic lesions in posterior descending artery (PDA) and posterior branches of left ventricle (PLV) of RCA

A typical case who underwent quantification analysis was showed in Fig. 5, and new quantitative parameters, such rMBF, sMBF, MFR and RFR, were obtained with the software of MyoFlowQ. Those quantitative parameters, such as sMBF, MFR and RFR, were very much abnormal, which indicated that this patient’s heart was in a very serious condition.

## Discussion

According to the literature search, the present study is the first to use low-dose CZT SPECT continuous dynamic tomography imaging to obtain some new quantitative parameters of MBF, such as sMBF, MFR and RFR, to evaluate and compare their correlation and diagnostic value in patients with suspected or known CAD. In this study, we found that for sMBF, MFR and RFR, no matter which reference standard (stenosis ≥ 50% or ≥ 75%) on CAG, the parameter values in the positive group were significantly lower than those in the negative group, and there was statistical difference. At the vessel level, no matter using stenosis ≥ 50% or ≥ 75% on CAG as the reference standard, there was a moderate to significant correlation among sMBF, MFR and RFR. In the four groups of non-obstructive atherosclerosis group, 1-vessel CAD group, 2-vessels CAD group and 3-vessels CAD group, there was no statistical difference between any two groups of LV-rMBF, suggesting that it did not have diagnostic value, while sMBF and MFR tended to decrease gradually, and there was a statistically significant difference between any two groups of LV-sMBF and LV-MFR (expect in 1-vessel CAD group vs 2-vessels CAD group and 2-vessels CAD group vs 3-vessels CAD group). Through the analysis of ROC curve, sMBF, MFR and RFR showed better diagnostic performance, of which sMBF was the most prominent, no matter which reference standard of stenosis ≥ 50% or ≥ 75% on CAG. When the patients with 3-vessels CAD were excluded, there was no statistical difference in diagnostic performance among the three parameters.

As we all know, conventional SPECT MPI plays an important role in non-invasive imaging diagnosis and evaluation of CAD [1–5]. However, some shortcomings of conventional SPECT MPI have also been reported, such as large imaging agent injection dose, long collection time, low image resolution, poor specificity of semi-quantitative analysis, and easy to underestimate or even miss diagnosis of LM and/or 3-vessels lesions [11, 12]. Therefore, how to make up for the shortcomings of conventional SPECT MPI has become a “hot point” of research. Luckily, the introduction of CZT SPECT provides great help to solve these problems, which is regarded as a “milestone” progress, and greatly improves the imaging performance of SPECT equipment [8–10]. PET/CT is considered as the “gold standard” in the application of quantitative MBF and its related parameters, but it is only applied in some large heart centers at present, and there are many limitations in clinical implementation, such as expensive equipment and drugs, limited installed capacity and passing capacity, etc. CZT SPECT has the ability of fast continuous tomographic imaging, convenient drug supply and relatively low-cost equipment. At the same time, the improvement of image sensitivity and resolution makes low-dose imaging easy to realize. Therefore, it has great hope in reducing radiation dose, rapid imaging, MBF quantification and clinical application. Recent studies [15, 16] suggest that compared with PET/CT MBF quantification, CZT SPECT has a better correlation with it, confirming its accuracy and convenience.

In general, when using SPECT MPI to diagnose CAD, one of the main factors affecting visual evaluation and semi-quantitative analysis is that we must choose a “normal” reference firstly, while the “normal” reference itself may not be “normal”. For example, (1) FOR the LM lesions, because the reduced blood flow is scattered in the LAD and LCX regions, compared with the “normal” RCA region, the semi-quantitative analysis leads to the underestimation of the lesion degree. If the RCA has obvious lesions at the same time, it may be further underestimated or even missed. (2) For 3-vessels lesions, the MBF in the 3-vessels regions has decreased in different degrees, while the regions with relatively low degree of perfusion defects are regarded as a “normal” reference, resulting in the extent of ischemia being underestimated. (3) If there are 3-vessels balanced lesions, due to the lack of contrast, it may lead to missed diagnosis. In this study, taking the cut-off values of MFR or sMBF obtained by ROC analysis as the diagnostic standard, the accuracy of judging 15 cases of 3-vessels lesions was 100% (15/15) and 73.3% (11/15), respectively, suggesting that the analysis of blood flow quantitative parameters has good value in multi-vessel lesions. In addition, due to the low energy of single-photon radionuclides, SPECT MPI artifacts caused by soft tissue attenuation have become a common problem, such as female dense tissue and/ or breast, as well as male diaphragm attenuation artifacts, is the common source of false positive diagnosis, but also the main reason for the poor specificity of conventional SPECT MPI diagnosis [19, 20]. MPI quantitative blood flow analysis plays an important role in overcoming the shortcomings of conventional SPECT imaging diagnosis because of complete attenuation and scattering correction and eliminating the effect of attenuation artifacts. The sMBF is the peak of MBF in the case of the peak effect of vasodilators, which is related to epicardial coronary artery dilatation and microcirculatory resistance.

Physiological experiments have confirmed that for the normal coronary artery system, the peak blood flow under drug-stress can be increased by 3 to 5 times as much as at rest. Gould et al. [21] first used experimental methods to verify the relationship between CFR (defined as MFR) and coronary artery stenosis, and the results showed that CFR was not affected when coronary stenosis was less than 50%, but decreased significantly in 50 to 95%, which was mainly caused by reduced stress blood flow, and pointed out that the stenosis on CAG could not directly predict the corresponding hemodynamic abnormalities. MFR is also an independent factor to predict the prognosis of patients, and the negative MFR of the global left ventricle has a better negative predictive value for high-risk patients [22]. Both Fame I and Fame II tests showed that the benefit of patients with coronary revascularization guided by FFR, was significantly better than that of CAG alone [23, 24].

RFR is defined by the ratio of a stenotic regional sMBF to normal perfusion regional sMBF obtained by quantitative MPI, which is considered to be similar to FFR, and can be used as an effective index to diagnose CAD [25]. Early studies [26] have shown that there is a good correlation between invasive FFR measured by pressure guide wire and RFR obtained by ^15^O-water PET (r = 0.87). At present, FFR measured by pressure guide wire has been used as an invasive “gold standard” to judge whether epicardial coronary artery stenosis affects myocardial perfusion. In this study, we found that sMBF, MFR and RFR in the positive group were lower than those in the negative group, and with the improvement of diagnostic criteria, there was a trend of further synchronous decrease, and the correlation among the three parameters was moderate or above. ROC curve analysis showed that the diagnostic efficiency of the three parameters was very strong, which was consistent with the results of previous studies [27, 28]. However, previous studies did not compare the correlation and diagnostic ability of sMBF, MFR and RFR at the same time, mostly analyzed one or two of them, and did not discuss the two commonly used CAD diagnostic criteria of stenosis ≥ 50% and 75% on CAG, respectively. The reference standard on CAG for clinical diagnosis of CAD is usually stenosis ≥ 50%, however, as the anatomical standard of interventional therapy or not, it is usually considered to be stenosis ≥ 70–80%. Therefore, the present study is divided into two situations and analyzed separately, in order to simulate the clinical actual situation. This is the innovation of this study design compared with previous studies, and we found that the diagnostic ability of sMBF is similar to MFR, when the 3-vessles CAD patients are removed, there is no significant difference in diagnostic ability among the three parameters, and it also suggests that RFR is not suitable for the diagnosis of 3-vessels lesions, because the reference regional sMBF is also decreased, resulting in no significant decrease in RFR. Conversely, sMBF and MFR are not affected by this, the wider the lesion is involved, the more obvious the decrease will be, which is confirmed in the analysis of grouping in this study. Therefore, sMBF and MFR have the same important values in the diagnosis and evaluation of obstructive CAD. It should be noted that for patients with negative CAG but with CMD, MPI tomographic images may be normal, while sMBF and CFR may be decreased [29]. At this point, if CAG is taken as the reference standard, the positive result of quantitative blood flow will be defined as false positive. Therefore, clinically suspected CMD was included in the exclusion criteria of this study.

### Study limitations

First, the sample size of this study is relatively small and due to the limitation of clinical technical accessibility, and it is not compared with the “gold” standard of FFR and index of microvascular resistance (IMR), which reflect epicardial coronary artery perfusion and microcirculation function. Second, obstructive CAD with microcirculation dysfunction cannot be distinguished by CZT SPECT quantitative parameters. Third, although clinically suspected CMD patients are enrolled in the exclusion criteria, due to the lack of IMR verification, it is not fully guaranteed whether CMD patients with atypical symptoms are included. Fourth, we admitted that although the recognized diagnostic cut-off value of MFR is 2.0–2.5, there is no unified diagnostic cut-off value for sMBF and RFR. Finally, due to different types of equipment and imaging agents, different physical correction factors, such as between CZT SPECT and PET/CT, between different types of CZT SPECT, between single-photon drugs and positron drugs, and between different single-photon drugs or positron drugs, the quantitative parameters obtained need to be further studied in detail.

## Conclusion

Our results demonstrate that new myocardial quantitative blood flow parameters of sMBF, MFR and RFR can be easily obtained by low-dose dynamic CZT SPECT, and the parameter values in the positive group were significantly lower than those in the negative group. There is a good correlation among the three parameters, which has a certain diagnostic values for patients with suspected or known CAD, and is a useful supplement to the conventional qualitative or semi-quantitative diagnostic methods.

## Data Availability

All data are fully available without restriction.

## Code availability

MyoFlowQ (Beijing Bailingyun Bio-Pharmaceutical Co., Ltd.).
